# Clinical Outcomes after Intracorporeal versus Extracorporeal Anastomosis in Patients Undergoing Laparoscopic Right Hemicolectomy for Colon Cancer

**DOI:** 10.3390/medicina60071073

**Published:** 2024-06-29

**Authors:** Yu-Yao Chang, Bill Cheng, Gwo-Tarng Sheu

**Affiliations:** 1 Institute of Medicine, Chung Shan Medical University, No. 110, Sec. 1, Jianguo N. Road, Taichung City 402, Taiwan; 177176@cch.org.tw; 2 Division of Colon and Rectal Surgery, Department of Surgery, Changhua Christian Hospital, 135 Nanhsiao Street, Changhua City 500, Taiwan; 3 Department of Post-Baccalaureate Medicine, College of Medicine, National Chung Hsing University, 145 Xingda Rd., Taichung City 402, Taiwan; 4 Graduate Institute of Biomedical Engineering, National Chung-Hsing University, Taichung City 402, Taiwan; bcheng@dragon.nchu.edu.tw; 5 Department of Medical Oncology and Chest Medicine, Chung Shan Medical University Hospital, No. 110, Sec. 1, Jianguo N. Road, Taichung City 402, Taiwan

**Keywords:** laparoscopic right hemicolectomy (LRHC), colon cancer, intracorporeal anastomosis (ICA), extracorporeal anastomosis (ECA), outcome

## Abstract

*Background and Objectives*: Laparoscopic right hemicolectomy (LRHC) is commonly performed for patients with colon cancer, selecting between intracorporeal anastomosis (ICA) or extracorporeal anastomosis (ECA). However, the impact of ICA versus ECA on patient outcomes remains debatable. The varying levels of experience among surgeons may influence the outcomes. Therefore, this study sought to compare the short- and long-term outcomes of LRHC using ICA versus ECA. *Materials and Methods*: This retrospective study extracted patient data from the medical records database of Changhua Christian Hospital, Taiwan, from 2017 to 2020. Patients with colon cancer who underwent LRHC with either ICA or ECA were included. Primary outcomes were post-surgical outcomes and secondary outcomes were recurrence rate, overall survival (OS), and cancer-specific survival (CSS). Between-group differences were compared using chi-square, *t*-tests, and Fisher’s exact tests and Mann–Whitney U tests. Associations between study variables, OS, and CSS were determined using Cox analyses. *Results*: Data of 240 patients (61 of ICA and 179 of ECA) with a mean age of 65.0 years and median follow-up of 49.3 months were collected. No recognized difference was found in patient characteristics between these two groups. The ICA group had a significantly shorter operation duration (median (IQR): 120 (110–155) vs. 150 (130–180) min) and less blood loss (median (IQR): 30 (10–30) vs. 30 (30–50) mL) than the ECA group (*p* < 0.001). No significant differences were found in 30-day readmission (ICA vs. ECA: 1.6% vs. 2.2%, *p* > 0.999) or recurrence (18.0% vs. 13.4%, *p* = 0.377) between the two groups. Multivariable analyses revealed no significant differences in OS (adjusted hazard ratio (aHR): 0.65; 95% confidence interval (CI): 0.25–1.44) or CSS (adjusted sub-hazard ratio (aSHR): 0.41, 95% CI: 0.10–1.66) between groups. *Conclusions*: Both ICA and ECA in LRHC for colon cancer had similar outcomes without statistically significant differences in post-surgical complications, 30-day readmission rates, recurrence rate, and long-term survival outcomes. However, ICA may offer two advantages in terms of a shorter operative duration and reduced blood loss.

## 1. Introduction

Colorectal cancer (CRC) is a significant health concern globally, and surgical resection remains the cornerstone in its management [1,2]. CRC incidence is decreasing among adults aged ≥50 years as a result of greater awareness among the public and medical professionals alike and availability of accessible screening tools, such as fecal tests, endoscopy, and computed tomography colonography [3,4,5]. Laparoscopic right hemicolectomy (LRHC) is a commonly performed procedure for patients with colon cancer [6,7]. Recent studies suggest that LRHC, which has gained popularity since its introduction in 1991 [8], offers advantages such as early recovery and fewer short-term complications compared with open right hemicolectomy. Moreover, evidence demonstrates that laparoscopic procedures, in general, provide superior short- and long-term safety and quality outcomes [9,10,11].

One critical aspect of LRHC is the choice of anastomotic technique, which can be either intracorporeal anastomosis (ICA) or extracorporeal anastomosis (ECA) [12,13,14]. However, the impact of this choice on patient outcomes, both in the short term and long term, remains an area of debate and investigation. The superiority of ICA, as indicated by a meta-analysis, suggests better postoperative outcomes and faster recovery, but the comparison between ICA and ECA remains a subject of controversy [15]. Therefore, this study aimed to compare short- and long-term outcomes of LRHC ICA with those with ECA, seeking to provide insights into the optimal approach for patients undergoing this procedure.

## 2. Materials and Methods

### 2.1. Study Design and Patient Selection

**Figure 1:**
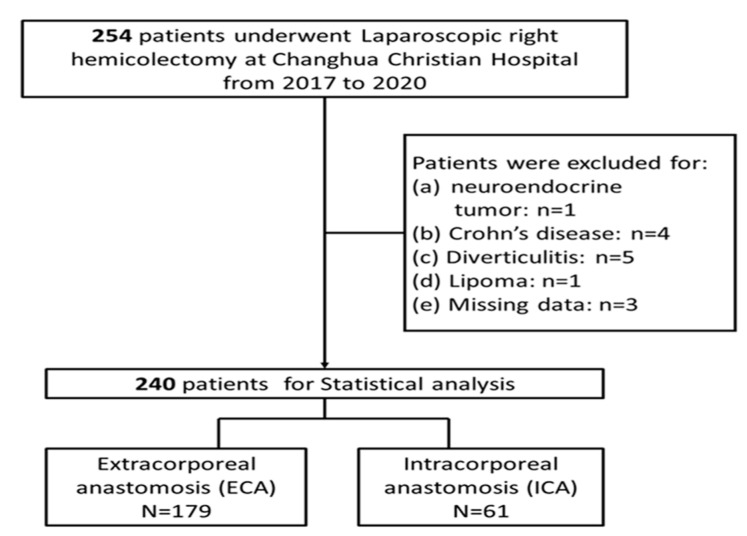
Flow chart of patient selection process.

This research was a retrospective, chart-review study, examining the institution-maintained database of a tertiary center to identify patients diagnosed with colon cancer who had undergone LRHC with either ICA or ECA at Changhua Christian Hospital, Taiwan. Consecutive patients who underwent LRHC from 2017 to 2020 were eligible for inclusion. Patients were excluded if they had a diagnosis of neuroendocrine tumor, underwent LRHC for a disease other than colon cancer, or had missing data on whether ICA or ECA were performed. Patients were then categorized into two distinct groups based on the specific anastomotic technique (ICA vs. ECA groups) employed (Figure 1). This study’s protocol was approved by the Institutional Review Board (IRB: CCH-IRB: 200306) of Changhua Christian Hospital, which waived the need for signed informed consent from the included patients since all data were de-identified.

### 2.2. Surgical Procedures

Four experienced senior surgeons at our institution, all skilled in laparoscopic colorectal surgery, conducted the procedures. One surgeon used the ICA technique, while the other three used ECA. Initially, both procedures involved creating pneumoperitoneum using the open Hasson technique. A 12 mm supra-umbilical camera port was used, along with 5 to 12 mm ports in the right flank, left upper quadrant, and left lower quadrant. The right colon was approached medially, and the ileocolic pedicle was ligated. In the ECA group, after mobilizing the hepatic flexure, right colon, and terminal ileum, a 5 cm vertical periumbilical incision was made. The bowel was extracted through a wound protector, resected using a linear stapler, and an extracorporeal hand-sewn, double-layer, end-to-end anastomosis was performed. A Jackson–Pratt drain tube was placed, and the incision was closed. In the ICA group, bowel resection was performed intracorporeally with an endoscopic linear stapler, creating a double-layer, isoperistaltic, side-to-side anastomosis. The enterocolotomy was closed with a continuous suture using a barbed knotless 3/0 suture. The specimen was extracted through a vertical periumbilical incision, and a Jackson–Pratt drain tube was placed similarly to the ECA group.

### 2.3. Study Variables and Outcomes

Patients’ characteristics included age, sex, BMI, and comorbidities, such as hypertension (HTN), cardiovascular disease (CVA), diabetes mellitus (DM), and end-stage renal disease (ESRD). Additionally, preoperative carcinoembryonic antigen (CEA), American Society of Anaesthesiologists (ASA) physical status, surgical type (scheduled or emergent), liver resection, tumor size, and staging were included. The primary outcomes were post-surgical outcomes, including operation duration, blood loss, postoperative CEA, complications, and 30-day readmission rate. Complications evaluated encompassed anastomotic leakage, surgical site infection, ileus, ureteral injury, and intraabdominal abscess, as well as severity of complication assessed by the Clavien–Dindo Classification [16]. Anastomotic leaks were classified into minor (managed without reoperation) and major (requiring clinical intervention). Ileus was defined as the absence of flatus by the fifth postoperative day, necessitating parenteral nutrition support. The secondary outcomes were recurrence rate, overall survival (OS), and cancer-specific survival (CSS).

### 2.4. Statistical Analysis

Descriptive statistics were utilized to present patient information, presented as counts (n) and percentages (%) for categorical data, and as the mean ± standard deviation (SD) or median with interquartile range (IQR: Q1–Q3) for continuous data based on normality assumption. Comparative analysis was conducted between ICA and ECA groups in terms of baseline characteristics, surgical outcomes, and long-term follow-up results. For categorical data, the chi-square test or Fisher’s exact test was employed, while the independent *t*-test or Mann–Whitney U test was used for continuous data.

For further analysis, a Cox Proportional Hazard (PH) model was employed to assess OS and identify potential prognostic factors. For CSS, deaths from other causes were treated as competing risks, and the Fine and Gray model [17] was employed. This model incorporates the concept of sub-distribution hazard to estimate the risk. The results should then display estimated hazard ratios for OS and estimated sub-hazard ratios for cancer-specific survival. Statistical analyses were performed using SAS software version 9.4 (SAS Institute Inc., Cary, NC, USA). All tests were two-sided, and a *p*-value of less than 0.05 was considered statistically significant.

## 3. Results

### 3.1. Patients

From the year 2017 to 2020, a total of 254 patients underwent LRHC for colon cancer at Changhua Christian Hospital, Taiwan. After excluding 1 case with neuroendocrine tumor, 4 cases with Crohn’s disease, 5 cases with diverticulitis, 1 case with lipoma, and 3 cases with missing data on ICA/ECA, data from a total of 240 patients were included for analysis, including 179 receiving ECA and 61 receiving ICA. The patients’ baseline characteristics are presented in Table 1. Of note, among the patients in the ECA group, five underwent simultaneous liver resection. These patients received laparoscopic wedge resection of the liver, not major resections. The procedures were performed by a skilled hepatic surgeon, with each procedure lasting for less than an hour and estimated blood loss under 30 mL. Therefore, we postulated that the liver resections did not significantly impact the short-term outcomes and the analyses.

Among all patients, the mean age was 65.9 (SD = 13.7) years and 51.7% were females. No statistical differences were found in baseline demographics (age, sex, BMI, and comorbidities) between the ECA and ICA groups. Regarding surgery-related variables, the proportion of emergent surgeries was 10.8%. The proportion of patients with advanced cancer stages (stage III or stage IV) was higher in the ECA group compared with the ICA group, with a statistically significant difference observed in the distribution of T-stage (*p* = 0.022). Additionally, the mean tumor size was significantly smaller in the ICA group (3.9 ± 2.3 cm) than that in the ECA group (4.8 ± 2.3 cm) (Table 1).

### 3.2. Postoperative Outcomes

The postoperative outcomes are shown in Table 2. No significant differences were shown in positive lymph nodes of the ECA group compared with those of the ICA group (*p* = 0.067). Meanwhile, the operation duration (median (IQR): 120 (110–155) vs. 150 (130–180) min) and blood loss (median (IQR): 30 (10–30) vs. 30 (30–50) mL) were significantly lower in the ICA group than in the ECA group (*p* < 0.001). Overall, 59 (24.6%) patients experienced postoperative complications, while no significant differences were observed between the ECA and ICA groups.

A total of 15 (6.3%), 26 (10.8%), and 26 (10.8%) patients had surgical site infection, ileus, and other complications, respectively, while no significant between-group differences were observed. In the ECA group, a total of eight (3.3%) cases had anastomotic leakage, one case had ureteral injury, and five cases had intraabdominal abscesses. The thirty-day readmission rates were 2.2% and 1.6% in the ECA and ICA groups, respectively, without statistically significant differences (*p* > 0.999) (Table 2).

### 3.3. Long-Term Outcomes

The long-term follow-up results are also shown in Table 2. The median follow-up duration for the entire cohort was 49.3 months. The recurrence rates were 18.0% and 13.4% in the ICA and the ECA group, respectively, without statistically significant differences (*p* = 0.377). Similarly, no statistically significant differences were found between the two groups in terms of all-cause mortality (*p* = 0.599) and cancer-specific mortality (*p* = 0.353) (Table 2).

### 3.4. Univariate Analysis of Factors Associated with OS and CSS

Associations between the study variables, OS and CSS, before adjustments for possible confounders are summarized in Table 3. Older age (HR = 1.05), with DM (HR = 1.87), postoperative CEA levels >5 ng/mL (HR = 4.92), as well as lymph nodes ratio (HR = 1.03), and presence of stage IV disease (HR = 17.91), were associated with an increased risk of all-cause mortality. The results of univariate analysis on CSS revealed that larger tumor size (sub-hazard ratio [SHR] = 1.14), postoperative CEA (SHR = 5.62), and lymph nodes ratio (SHR = 1.05) were significantly associated with an increased risk of cancer-specific mortality (Table 3).

### 3.5. Multivariable Analysis of Factors Associated with OS and CSS

Associations between the study variables, OS, and CSS, after adjustments for possible confounders in the multivariable analyses are summarized in Table 4. Older age (aHR = 1.04), higher pathological stage (III and IV vs. 0–II, aHR = 3.99), and lymph node ratio (aHR = 1.03) were significantly associated with a higher risk of overall mortality. However, after adjusting for some variables, which were significant in univariate analysis, only the lymph node ratio remained significantly associated with a greater risk of cancer-specific mortality. No significant differences were found in OS (aHR: 0.65 95% CI: 0.25–1.44) and CSS (aSHR: 0.41, 95% CI: 0.10–1.66) between ICA versus ECA (Table 4).

## 4. Discussion

This study revealed that, during LRHC performed for colon cancer resection, ICA resulted in a shorter operation duration and lower blood loss compared with ECA, despite the minimal differences. Moreover, no statistically significant differences were found in postoperative complications and the 30-day readmission ratio between patients undergoing ICA or those undergoing ECA. Further, long-term outcomes, including recurrence rates and survival, also showed no significant disparities between the ICA and ECA groups. In summary, these results suggest that the clinical outcomes of ICA and ECA for LRHC are comparable, although ICA may be a potentially more favorable choice in the context of shorter operative time and less blood loss.

Both the advantages and disadvantages associated with ICA and ECA have been documented in the literature. In ECA, the practice of exteriorizing the specimen provides the surgical team with the opportunity for thorough visual inspection and palpation before resection and anastomosis procedures are initiated [12,18]. Accordingly, ECA is noted for its potential to mitigate the risk of intra-abdominal spillage of colonic contents during the surgical process [12]. However, it is imperative to acknowledge that the technique of exteriorizing the bowel for resection and anastomosis in ECA necessitates the mobilization of a significantly larger portion of the bowel and mesentery to achieve the requisite reach for specimen resection and subsequent anastomosis. This heightened mobilization poses a spectrum of potential complications, including an increased susceptibility to infections, traction injuries to the bowel or mesentery, serosal injuries, mesenteric bleeding, and the risk of devascularization of both the bowel and mesentery. These complications, in turn, may contribute to the occurrence of postoperative ileus [13,19]. Conversely, the dynamics of ICA present a contrasting set of advantages and disadvantages. Notably, the absence of the need for bowel and mesentery retraction in ICA minimizes the risk of traction injuries. This reduction in mechanical trauma not only lowers the likelihood of immediate complications, but also has a positive downstream effect, potentially reducing the incidence of incisional hernias and mitigating associated long-term morbidity [20,21].

In this study, a significantly shorter operation duration was reported for ICA compared with ECA, which was aligned with the results of previous studies [22,23,24]. Although the estimated intraoperative blood loss was seldom reported in other studies, researchers have documented that the length of stay was shorter in ICA than ECA [23,24,25,26,27,28,29]. Moreover, in line with our findings, most studies in the literature reported that the readmission rate [22,24,30], recurrence rate [22,31], OS [23,27,31], and CSS [23,28] were comparable between ICA and ECA groups.

Sun et al. [32] reported that surgical infection was higher in patients with ICA, and that patients receiving ICA had higher risk of Clavien-Dindo grade I and II postoperative complications than those who received ECA. Since the surgical site was inside the body, the intra-abdominal spillage of colonic contents during the surgical process may be responsible for this difference. Nevertheless, this difference was not observed in those with grade III or those with higher complications [32]. In the present study, neither surgical site infection nor complications graded by the Clavien–Dindo classification were significantly different between ICA and ECA.

For long-term survival outcomes, our results demonstrated several predictors for poor OS and CSS. Specifically, older age, advanced cancer stage, and greater lymph node ratio independently predicted an increased risk of poor OS, while a greater lymph node ratio was associated with an increased risk of poor CSS. This is generally consistent with current knowledge [33,34].

CEA is commonly used for predicting the prognosis of colorectal cancer. A CEA level higher than 5 ng/mL is generally associated with a shorter recurrence-free interval, reduced CSS and OS [35,36]. The results of previous studies have also suggested that both elevated pre-operative and post-surgical CEA were associated with poor survival outcomes [37]. The results of the present study did not show significant association between postoperative CEA > 5 ng/mL and the risk of poorer OS and CSS, despite the trends observed. This could be interpreted in the context of insufficient statistical power, differences in patient characteristics, or the variations in the timing of CEA measurements post-surgery, all of which might have affected the expected impact of CEA on survival outcomes.

## 5. Limitations

The present study has certain limitations that must be acknowledged. First, the retrospective nature of this study hinders its ability to provide causal inferences and may be subject to selection bias. Second, the sample size of this study was relatively small, which may have affected the power of the statistical analyses to detect the differences. Third, the ICA technique was administered exclusively by a single surgeon, whereas the ECA technique was carried out by three different surgeons. The varying levels of experience among these surgeons may influence the analytical outcomes. Additionally, among the methodological limitations of the study are the different techniques used to perform the anastomoses. In our study, ICAs were mechanical latero–lateral anastomoses, whereas ECAs were manual end-to-end anastomoses. This difference in technique might have influenced the outcomes observed in this study. Additional prospective studies with a larger sample size are still warranted to confirm the present findings.

## 6. Conclusions

In conclusion, the results of this study indicate that ICA in LRHC for colon cancer may have advantages in terms of a shorter operative duration and reduced blood loss compared with those of ECA. However, no significant differences were seen in postoperative complications, 30-day readmission rates, and long-term survival between the two procedures. ICA can be a favorable option for surgeons, particularly when it aligns with their surgical skills and preferences. Nevertheless, such a decision should still be made with careful consideration of the individual patient’s characteristics and the surgeon’s expertise in order to ensure optimal patient care. Given the limited sample size, the analyses may be under-powered. Future studies with larger sample sizes are necessary to verify the findings and address the study question more conclusively.

## Figures and Tables

**Table 1:** Patients’ baseline characteristics.

	All Patients	ICA	ECA	*p*-Value
	n = 240	n = 61	n = 179	
Age, years	65.9 ± 13.7	65.6 ± 14.3	65.9 ± 13.6	0.867
Sex				0.455
Female	124 (51.7)	29 (47.5)	95 (53.1)	
Male	116 (48.3)	32 (52.5)	84 (46.9)	
BMI, kg/m^2^	24.6 ± 4.3	25.2 ± 5.5	24.3 ± 3.9	0.238
Comorbidities				
DM	51 (21.3)	15 (24.6)	36 (20.1)	0.460
HTN	86 (35.8)	25 (41.0)	61 (34.1)	0.331
ESRD	7 (2.9)	2 (3.3)	5 (2.8)	>0.999
CVA	10 (4.2)	3 (4.9)	7 (3.9)	0.717
Preoperative CEA (ng/mL) *	3 (1.9–8.8)	2.85 (1.9–8.65)	3.2 (1.8–8.8)	0.762
<=5	148 (0.6)	39 (0.7)	109 (0.6)	0.707
>5	87 (0.4)	21 (0.4)	66 (0.4)	
ASA				0.204
I	9 (3.8)	0 (0.0)	9 (5.0)	
II	101 (42.1)	28 (45.9)	73 (40.8)	
III	130 (54.2)	33 (54.1)	97 (54.2)	
Surgical Type				0.443
Scheduled	214 (89.2)	56 (91.8)	158 (88.3)	
Emergent	26 (10.8)	5 (8.2)	21 (11.7)	
Site				0.097
Appendix	16 (6.7)	7 (11.5)	9 (5.0)	
Cecum	41 (17.1)	14 (23.0)	27 (15.1)	
Ascending colon	118 (49.2)	28 (45.9)	90 (50.3)	
Transverse colon	65 (27.1)	12 (19.7)	53 (29.6)	
With liver resection	5 (2.1)	0 (0.0)	5 (2.8)	0.333
T stage				0.022
T0	31 (12.9)	13 (21.3)	18 (10.1)	
T1	22 (9.2)	8 (13.1)	14 (7.8)	
T2	31 (12.9)	11 (18.0)	20 (11.2)	
T3	132 (55.0)	25 (41.0)	107 (59.8)	
T4	24 (10.0)	4 (6.6)	20 (11.2)	
N stage				0.130
N0	153 (63.8)	45 (73.8)	108 (60.3)	
N1	52 (21.7)	11 (18.0)	41 (22.9)	
N2	35 (14.6)	5 (8.2)	30 (16.8)	
M stage				0.281
M0	211 (87.9)	56 (91.8)	155 (86.6)	
M1	29 (12.1)	5 (8.2)	24 (13.4)	
Pathologic stage				0.056
0	31 (12.9)	13 (21.3)	18 (10.1)	
I	47 (19.6)	16 (26.2)	31 (17.3)	
II	67 (27.9)	14 (23.0)	53 (29.6)	
III	66 (27.5)	13 (21.3)	53 (29.6)	
IV	29 (12.1)	5 (8.2)	24 (13.4)	
Tumor size, cm	4.6 ± 2.4	3.9 ± 2.3	4.8 ± 2.3	0.009

* 5 cases were missing. Mean ± SD or median (IQR) are presented for continuous variables, and n (%) for categorical variables. ASA, American Society of Anesthesia; ICA, intracorporeal anastomosis; ECA, extracorporeal anastomosis; BMI, body mass index; DM, diabetes mellitus; HTN, hypertension; ESRD, end-stage renal disease; CVA, cerebrovascular accident; CEA, carcinoembryonic antigen; *p*-values < 0.05 are shown in bold.

**Table 2:** Postoperative and long-term outcomes.

	All Patients	ICA	ECA	*p*-Value
	n = 240	n = 61	n = 179	
Postoperative outcomes				
Number of positive lymph nodes				0.067
0	156 (65.0)	47 (77.0)	109 (60.9)	
1–3	49 (20.4)	9 (14.8)	40 (22.3)	
≥4	35 (14.6)	5 (8.2)	30 (16.8)	
Number of harvested lymph nodes				0.492
<12	7 (2.9)	1 (1.6)	6 (3.4)	
≥12	233 (97.1)	60 (98.4)	173 (96.6)	
Lymph node ratio	0.07 ± 0.17	0.04 ± 0.14	0.08 ± 0.17	0.070
Operation duration, min	150 (120–180)	120 (110–155)	150 (130–180)	<0.001
Estimated blood loss, mL	30 (30–50)	30 (10–30)	30 (30–50)	<0.001
Postoperative CEA (ng/mL) *	2.3 (1.6–4)	2 (1.7–3.4)	2.35 (1.6–4.1)	0.566
≤5	175 (86.2)	42 (93.3)	133 (84.2)	0.116
>5	28 (13.8)	3 (6.7)	25 (15.8)	
Complication	59 (24.6)	16 (26.2)	43 (24.0)	0.730
Anastomotic leakage	8 (3.3)	0 (0.0)	8 (4.5)	0.208
Minor/Major	6/2	0/ 0	6/2	
Surgical site infection	15 (6.3)	3 (4.9)	12 (6.7)	0.766
Minor/need operation	7/ 8	1/ 2	6/ 6	
Ileus	26 (10.8)	4 (6.6)	22 (12.3)	0.213
Ureteral injury	1 (0.4)	0 (0.0)	1 (0.6)	>0.999
Intraabdominal abscess	5 (2.1)	0 (0.0)	5 (2.8)	0.333
Other complications	26 (10.8)	10 (16.4)	16 (8.9)	0.106
Clavien–Dindo Classification				0.698
None	181 (75.4)	45 (73.8)	136 (76.0)	
Grade I	17 (7.1)	4 (6.6)	13 (7.3)	
Grade II	24 (10.0)	8 (13.1)	16 (8.9)	
Grade III	13 (5.4)	2 (3.3)	11 (6.1)	
Grade IV	0 (0.0)	0 (0.0)	0 (0.0)	
Grade V	5 (2.1)	2 (3.3)	3 (1.7)	
30-day readmission	5 (2.1)	1 (1.6)	4 (2.2)	>0.999
Long-term outcomes				
Follow-up, months	49.3 (38.4–60.0)	51.3 (37.8–59.0)	48.4 (38.7–60.7)	0.967
Recurrence	35 (14.6)	11 (18.0)	24 (13.4)	0.377
All-cause death	53 (22.1)	12 (19.7)	41 (22.9)	0.599
Cancer-specific death	23 (9.6)	4 (6.6)	19 (10.6)	0.353

* 37 cases were missing. Median (IQR) is presented for continuous variables and n (%) for categorical variables. ICA, intracorporeal anastomosis; ECA, extracorporeal anastomosis; CEA, carcinoembryonic antigen, *p*–values < 0.05 are shown in bold.

**Table 3:** Univariate analysis on the factors associated with OS and CSS.

	OS		CSS	
	Crude HR (95%CI)	*p*-Value	Crude SHR (95%CI)	*p*-Value
Anastomosis (ICA vs. ECA)	0.91 (0.46–1.69)	0.778	0.62 (0.21–1.78)	0.370
Age, years	1.05 (1.03–1.08)	<0.001	1.01 (0.98–1.04)	0.588
Sex (male vs. female)	1.05 (0.61–1.81)	0.865	0.44 (0.18–1.07)	0.071
BMI	0.99 (0.92–1.05)	0.718	1.03 (0.94–1.12)	0.550
DM	1.87 (1.01–3.32)	0.038	1.37 (0.54–3.53)	0.508
HTN	1.51 (0.87–2.61)	0.138	0.97 (0.41–2.30)	0.946
ESRD	2.19 (0.53–5.97)	0.188	1.45 (0.18–11.44)	0.722
CVA	2.53 (0.76–6.23)	0.075	1.22 (0.15–9.74)	0.850
Anatomic Site				
Appendix	Ref			
Ascending colon	0.93 (0.32–3.95)	0.910	0.73 (0.16–3.25)	0.677
Cecum	1.12 (0.33–5.06)	0.862	0.53 (0.09–3.08)	0.477
Transverse colon	1.30 (0.44–5.58)	0.671	0.63 (0.13–3.09)	0.572
pStage			NA	
0	Ref			
I	1.58 (0.33–11.35)	0.595		
II	1.97 (0.50–13.06)	0.389		
III	4.11 (1.12–26.80)	0.067		
IV	17.91 (4.99–116.0)	<0.001		
Tumor size, cm	1.10 (0.99–1.22)	0.070	1.14 (1.03–1.27)	0.011
Post-op CEA (>5 vs. ≤5 ng/mL)	4.92 (2.43–9.50)	<0.001	5.62 (2.31–13.69)	<0.001
Complication	2.09 (1.18–3.64)	0.010	1.35 (0.55–3.35)	0.512
Lymph node ratio	1.03 (1.02–1.04)	<0.001	1.05 (1.04–1.06)	<0.001

ICA, intracorporeal anastomosis; ECA, extracorporeal anastomosis; BMI, body mass index; DM, diabetes mellitus; HTN, hypertension; ESRD, end-stage renal disease; CVA, cerebrovascular accident; CEA, carcinoembryonic antigen; pStage, pathological cancer stage; OS, overall survival; CSS, cancer-specific survival; HR, hazard ratio; SHR, sub-hazard ratio; CI, confidence interval; Ref, reference; NA, not applicable. *p*–value < 0.05 are shown in bold. NA: the estimates are not applicable due to strong correlation between stage and cancer-specific death. Estimates diverge. Lymph node ratio: Estimate by per 0.01 unit change.

**Table 4:** Multivariable analysis on the factors associated with OS and CSS.

**OS**	**aHR (95%CI)**	***p*-Value**
Anastomosis (ICA vs. ECA)	0.65 (0.25–1.44)	0.378
Age	1.04 (1.01–1.07)	**0.005**
DM	0.92 (0.42–1.87)	0.821
pStage (III–IV vs. 0–II) *	3.99 (1.68–10.57)	**0.002**
Post-OP CEA (>5 vs. ≤5 ng/mL)	1.95 (0.80–4.39)	0.091
Complication (Yes vs. No)	1.17 (0.53–2.45)	0.668
Lymph node ratio	1.03 (1.02–1.04)	**<0.001**
**CSS**	**aSHR (95%CI)**	***p*-Value**
Anastomosis (ICA vs. ECA)	0.41 (0.10–1.66)	0.211
Tumor size	0.98 (0.79–1.23)	0.885
Post-OP CEA (>5 vs. ≤5 ng/mL)	1.75 (0.51–6.08)	0.375
Lymph node ratio	1.05 (1.04–1.07)	**<0.001**

CEA, carcinoembryonic antigen; DM, diabetes mellitus; ICA, intracorporeal anastomosis; ECA, extracorporeal anastomosis; pStage, pathological cancer stage; CI, confidence interval; Ref, reference; OS, overall survival; CSS, cancer-specific survival; CEA, carcinoembryonic antigen; aHR, adjusted hazard ratio; aSHR, adjusted sub-hazard ratio. * Several stages were combined for analysis in case of estimated diverge. *p*-values < 0.05 are shown in bold. Lymph node ratio: Estimate by per 0.01 unit change.

## Data Availability

The data used and/or analyzed during the current study are available from the corresponding author on reasonable request.
